# Selective optogenetic stimulation of efferent fibers in the vagus nerve of a large mammal

**DOI:** 10.1016/j.brs.2020.11.010

**Published:** 2021

**Authors:** Lindsea C. Booth, Song T. Yao, Alla Korsak, David G.S. Farmer, Sally G. Hood, Daniel McCormick, Quinn Boesley, Angela A. Connelly, Stuart J. McDougall, Willian S. Korim, Sarah-Jane Guild, Svetlana Mastitskaya, Phuong Le, Anja G. Teschemacher, Sergey Kasparov, Gareth L. Ackland, Simon C. Malpas, Robin M. McAllen, Andrew M. Allen, Clive N. May, Alexander V. Gourine

**Affiliations:** a Florey Institute of Neuroscience and Mental Health, University of Melbourne, Melbourne, Australia; b Florey Department of Neuroscience and Mental Health, MDHS, University of Melbourne, Melbourne, Australia; c Centre for Cardiovascular and Metabolic Neuroscience, Department of Neuroscience, Physiology and Pharmacology, University College London, London, UK; d Department of Physiology and Auckland Bioengineering Institute, University of Auckland, Auckland, New Zealand; e Department of Physiology, The University of Melbourne, Melbourne, Australia; f Physiology, Neuroscience and Pharmacology, University of Bristol, Bristol, UK; g Baltic Federal University, Kaliningrad, Russian Federation; h Translational Medicine and Therapeutics, William Harvey Research Institute, Queen Mary University of London, London, UK

**Keywords:** Autonomic nervous system, Brainstem, Neuromodulation, Optogenetic, Vagal preganglionic neurons, Vagus nerve stimulation, DVMN, dorsal motor nucleus of the vagus nerve, LED, light emitting diode, LVV, lentiviral vector, optoVNS, optogenetic vagus nerve stimulation, PCO2, partial pressure of carbon dioxide, PO2, partial pressure of oxygen, VNS, vagus nerve stimulation

## Abstract

**Background:**

Electrical stimulation applied to individual organs, peripheral nerves, or specific brain regions has been used to treat a range of medical conditions. In cardiovascular disease, autonomic dysfunction contributes to the disease progression and electrical stimulation of the vagus nerve has been pursued as a treatment for the purpose of restoring the autonomic balance. However, this approach lacks selectivity in activating function- and organ-specific vagal fibers and, despite promising results of many preclinical studies, has so far failed to translate into a clinical treatment of cardiovascular disease.

**Objective:**

Here we report a successful application of optogenetics for selective stimulation of vagal efferent activity in a large animal model (sheep).

**Methods and results:**

Twelve weeks after viral transduction of a subset of vagal motoneurons, strong axonal membrane expression of the excitatory light-sensitive ion channel ChIEF was achieved in the efferent projections innervating thoracic organs and reaching beyond the level of the diaphragm. Blue laser or LED light (>10 mW mm^−2^; 1 ms pulses) applied to the cervical vagus triggered precisely timed, strong bursts of efferent activity with evoked action potentials propagating at speeds of ∼6 m s^−1^.

**Conclusions:**

These findings demonstrate that in species with a large, multi-fascicled vagus nerve, it is possible to stimulate a specific sub-population of efferent fibers using light at a site remote from the vector delivery, marking an important step towards eventual clinical use of optogenetic technology for autonomic neuromodulation.

## Highlights

• Described is a method of selective efferent vagus nerve stimulation using light.
• Vagal preganglionic neurons are targeted to express light-sensitive channels.
• Specific efferent VNS by light delivery to the cervical vagus is achieved in a large animal model.
• Demonstrates feasibility of using optogenetic technology for autonomic neuromodulation.

## Introduction

Electroceuticals are devices that treat medical conditions by application of electrical stimulation to individual peripheral nerves, specific brain regions, organs, or transcutaneously. Therapeutic electroceutical treatments have been trialed in a range of conditions characterized by autonomic dysfunction, including heart failure, hypertension, autoimmune and inflammatory diseases [1]. One such treatment, vagus nerve stimulation (VNS), was developed with the aim of redressing the autonomic imbalance in cardiovascular disease by increasing the activity of the parasympathetic arm of the autonomic nervous system, via application of electrical current pulses to the vagus nerve at the cervical level [[2], [3], [4], [5]]. However, the promising results obtained with this approach in preclinical studies failed to translate to the clinic, likely due to the complexity of the vagus nerve and the non-selective nature of electrical VNS. For example, large trials of electrical VNS in hundreds of heart failure patients failed to demonstrate benefits with respect to the primary endpoints despite strong pre-clinical data [6].

The vagus nerve is a mixed nerve that innervates the majority of the viscera and contains afferent sensory and efferent motor vagal fibers at an approximate ratio of 4:1 as well as sparse sympathetic fibers [[7], [8], [9]]. Electrical stimulation of mixed nerves does not discriminate between the various subsets of afferent and efferent fibers, nor between the functional subtypes of each of these groups of fibers. Stimulation of the cervical vagus (a common site for VNS due to the relative ease of access) preferentially activates sensory fibers as they have a lower activation threshold than the efferent fibers [10]. As such, stimulations with currents that are sufficient to activate efferent fibers also capture vagal afferents, resulting in undesirable side effects [11,12]. To increase the efficacy of VNS while minimizing the side effects, the development of novel methods allowing selective stimulation of specific functional groups of vagal fibers is highly desirable.

The required level of selectivity can be achieved by transducing functional populations of vagal neurons to express native or modified light-sensitive ion channels derived from algae [13]. This approach allows stimulation of electrical activity using light and had become a mainstream tool in neuroscience called optogenetics [13,14]. Several reports have demonstrated the use of optogenetic tools to stimulate peripheral nerves in rodents, some involving wireless technologies [[15], [16], [17], [18], [19], [20], [21], [22], [23]]. However, if optogenetic techniques are to be translated to the clinic, the feasibility and efficacy of using light to stimulate neuronal pools or select fibers in peripheral nerves need to be demonstrated in clinically relevant large animal models.

Here we report the successful application of optogenetics for selective stimulation of the efferent axons of vagal preganglionic neurons, whose somata are located in the dorsal motor nucleus of the vagus nerve (DVMN) in sheep. We show that 12 weeks after viral transduction of a subset of vagal motoneurons, strong axonal membrane expression of the excitatory light-sensitive ion channel ChIEF is achieved in the projections innervating thoracic organs and reaching beyond the level of the diaphragm. This allows selective stimulation of vagal efferent fibers using light (optoVNS) applied to the nerve at the cervical level or near the target organs.

## Materials and methods

### Genetic targeting of vagal preganglionic neurons

Vagal preganglionic neurons of the DVMN were transduced to express the light-sensitive channel ChIEF fused to the fluorescent protein tdTomato (ChIEF-tdTomato) [24]. ChIEF is a chimeric channelrhodopsin derivative constructed from the N-terminal part of channelrhodopsin 1 and the C-terminal part of channelrhodopsin 2. ChIEF combines the reduced inactivation characteristics of channelrhodopsin 1 with the more favorable cation permeability properties of channelrhodopsin 2 [24]. The I170V mutation further improves the channel closure kinetics. In comparison to channelrhodopsin 2, ChIEF produces greater photocurrents, allows better temporal control of neuronal activity by light pulses, and shows more efficient membrane expression and trafficking [24].

Vagal preganglionic neurons characteristically express the transcriptional factor Phox2 allowing selective targeting of these neurons using viral vectors driving the transgene expression under the control of the PRSx8 promoter constructed by multimerization of the Phox2 binding sites [25]. In the dorsal brainstem, only the DVMN neurons, a subset of neurons in the neighbouring nucleus of the solitary tract and sparse neurons of the area postrema express Phox2 transcription factors. Therefore, the PRSx8 promoter can only be active in these populations of cells. However, it appears that not all cells which express Phox2 efficiently drive the PRSx8 promoter. In our previous studies in rats we observed the strongest PRSx8-driven transgene expression in the DVMN with only sparse labelling in neurons of the adjacent areas [[26], [27], [28], [29]].

Lentiviruses were derived from the four-plasmid (pTYF transducing, pNHP packaging, VSVg envelope and pTAT plasmids) HIV-1-based self-inactivating lentiviral vector (LVV) system, pseudo-typed with the VSV-G envelope [30,31]. A detailed description of the methods used to generate and characterize these vectors has been reported previously [31,32]. Transgene expression driven under the control of the PRSx8 promoter was enhanced by the chimeric transactivator consisting of a GAL4-binding domain fused to a part of the transcriptional activation domain of NF-kBp65, and the multimerized GAL4-binding sequences (G4BS), as described [33]. The titer of the virus was 7.7 × 10^10^ IU ml^−1^, determined by qPCR performed on transduced cells, as described [34].

Validation of this vector system in targeting the DVMN neurons has been described in detail previously [26,28]. Single-cell recordings in rat brainstem slices demonstrated that 445–475 nm light triggered precisely timed depolarizations and action potential firing in DVMN neurons transduced to express ChIEF [26].

### Experiments in rats

Validation experiments were first performed in young adult male Sprague-Dawley rats (200–250 g; n = 5). The study was conducted in accordance with the European Commission Directive 2010/63/EU (European Convention for the Protection of Vertebrate Animals used for Experimental and Other Scientific Purposes) and the UK Home Office (Scientific Procedures) Act (1986) with project approval from the University College London Institutional Animal Care and Use Committee. The rats were group-housed and maintained on a 12-h light cycle (lights on 07:00) and had *ad libitum* access to water and food.

Rats were anaesthetized with ketamine (60 mg kg^−1^; i.m.) and medetomidine (250 μg kg^−1^; i.m.). Adequate depth of surgical anesthesia was maintained and confirmed by the absence of a withdrawal response to a paw pinch. The animal was placed in a stereotaxic frame and a midline dorsal neck incision was made to expose the dorsal brainstem surface. One microinjection of LVV-PRSx8-ChIEF-tdTomato (0.5 μl at a rate of 0.1 μl min^−1^) was made to the right DVMN using the following coordinates: 0.5 mm rostral, 0.6 mm lateral and 0.8 mm ventral from the *calamus scriptorius*. After the microinjections, the wound was sutured and anesthesia was reversed with atipamezole (1 mg kg^−1^, i.m.). For postoperative analgesia the animals received buprenorphine (0.05 mg kg^−1^ per day, s.c.) for 3 days. No complications were observed after the surgery and the animals gained weight normally.

Four weeks after microinjections of the viral vector, rats were anaesthetized with urethane (induction: 1.3 g kg^−1^, i.p.; maintenance: 10–25 mg kg^−1^ h^−1^, i.v.) and instrumented as described in detail previously [35,36]. Adequate depth of anesthesia was monitored and confirmed by the stability of arterial blood pressure and heart rate recordings which did not show responses to a paw pinch. The femoral artery and vein were cannulated for the measurements of blood pressure and the administration of anesthetic, respectively. The animal was intubated and mechanically ventilated with oxygen-supplemented air using a small rodent ventilator (tidal volume ∼1 mL per 100 g of body weight; ∼60 strokes min^−1^). PO_2_, PCO_2_, and pH of the arterial blood were measured regularly and kept within physiological ranges (PO_2_ 95–105 mmHg; PCO_2_ 35–45 mmHg and pH 7.35–7.45) by adjusting the tidal volume and/or ventilator frequency as well as the level of supplemental oxygen. Body temperature was maintained at 37.0 ± 0.5 °C. The animal was placed in a stereotaxic frame and the dorsal brainstem was exposed. For optical stimulation, laser light (445 nm) was delivered to the dorsal brainstem surface via an optrode with a diameter of 0.2 mm (Art Photonics, Berlin, Germany). The right vagus nerve was dissected, isolated from the surrounding tissues, placed on silver wire recording electrodes and covered with dental impression material. The recorded signal was amplified ( × 10,000), filtered (80–1500 Hz), and sampled at a rate of 5 kHz.

### Experiments in sheep

The experiments were performed in accord with the National Health and Medical Research Council of Australia guidelines and were approved by the Animal Ethics Committee of the Florey Institute of Neuroscience and Mental Health. Merino ewes (1–2 years old; 35–45 kg body weight) were group-housed on a natural light cycle and were given free access to water and 800 g oaten chaff per day. The animals were fasted on the day before the surgery.

Anesthesia was induced with sodium thiopentone (15 mg kg^−1^, i.v.) and, after endotracheal intubation, was maintained with 1.5–2.0% isoflurane in oxygen-supplemented air. Sheep were then secured in a custom-built stereotaxic frame. Adequate depth of surgical anesthesia was maintained and confirmed by lack of a corneal reflex. The animals were treated with intramuscular antibiotic (penicillin, 900 mg) at surgery and for 2 days post-operatively. Analgesia was maintained with intramuscular flunixin meglumine (1 mg kg^−1^) at surgery and for 4-h post-surgery. After incision of the overlying skin, the nuchal muscles were dissected, and occipital bone was partially removed to expose the caudal part of the cerebellum. The cerebellum was carefully raised to expose the fourth ventricle and the area postrema. Neurons of the left DVMN were targeted to express ChIEF-tdTomato. Three microinjections (3 μl each) of LVV-PRSx8-ChIEF-tdTomato were placed 2 mm below the surface of the brainstem at the mid-point of area postrema (1 mm lateral from the midline), at the level of the obex (2 mm lateral) and 2 mm caudal from obex (2 mm lateral). The micropipette was left in place for 5 min after each microinjection. At the end of the surgery, the dura, nuchal muscles and skin were separately sutured and the sheep were weaned off anesthesia. No complications were observed after the surgery.

After a minimum period of 9 weeks (average 12 ± 2 weeks, range 9–17 weeks), to allow for a high and stable level of ChIEF-tdTomato expression in the DVMN cell bodies and axons, sheep were anaesthetized as described above. Adequate depth of anesthesia was ensured by lack of a corneal reflex. The animal was intubated and mechanically ventilated with oxygen-supplemented air (tidal volume 500 mL; ∼12 strokes min^−1^). The body temperature was maintained at ∼38 °C and oxygen saturation was monitored. The left cervical vagus nerve was dissected, isolated from the surrounding tissues and sectioned. The distal end of the vagus nerve was then separated into four fascicle bundles. A small section of each of the bundles was removed and examined under the microscope to assess the expression of tdTomato in vagal fibers. The fascicle bundle showing the highest level of tdTomato expression was placed over a pair of silver wire hook electrodes submerged in a paraffin oil pool made by suturing of the surrounding skin. An earth electrode was placed against the muscle near the recording site. The vagal efferent activity was amplified ( × 2000), filtered (1–100 Hz), and sampled at a rate of 5 kHz.

For optical stimulation of the DVMN axons at the cervical level, the proximal region (55–70 mm from the recording electrodes) of the nerve was illuminated with either blue laser light (473 nm, 12 mW) via an optic fiber (1 mm in diameter) or an LED (Cree XPE2 Blue 475 nm; for light intensity measurement and calibration see Supplementary Text and Supplementary Table 1). Pulses of light (1, 2, and 5 ms) were delivered at a frequency of 1 Hz for 300 s. In one animal, the head was secured in a stereotaxic frame and the dorsal brainstem was exposed to allow stimulation of the DVMN cell bodies by application of laser light (473 nm) delivered to the dorsal brainstem surface via an optic fiber (1 mm in diameter). In another preparation, in addition to stimulation and recording of the cervical vagus nerve, a recording was made in the thoracic region of the vagus just proximal to the cardiac branch. The fifth rib was removed, and the vagus nerve in the thoracic cavity was identified, isolated, and placed on the recording electrodes. Light stimulation of the nerve was applied at the cervical level using the LED (1 Hz, 5 ms pulses, 300 s). In one animal, the cervical vagus was stimulated using electrical current pulses (0.2–20 V, 1–2 ms, 1 Hz) with recordings made 70 mm distal to the stimulating electrodes, as described [37].

### Histology

At the end of the experiments, the animals were given a terminal dose of pentobarbitone sodium (100 mg kg^−1^, i.v.). After perfusion via the carotid arteries with 0.9% saline followed by ice-cold 4% (w/v) paraformaldehyde, the brain was removed. The cervical vagus nerve, the vagus nerve proximal to the cardiac branch, the cardiac branch of the vagus nerve, the vagus nerve distal to the cardiac branch and the nodose ganglia were removed and fixed in 4% paraformaldehyde in phosphate-buffered saline (0.1 M, pH 7.4) for 24–48 h. After cryoprotection (20% sucrose), the tissues were frozen and sectioned (30 μm) to determine the extent of ChIEF-tdTomato expression by the DVMN neuronal cell bodies and projecting axons. Sections of the nerve were stained for Protein Gene Product 9.5 (PGP9.5) using rabbit anti-Human PGP9.5 antibody (Cedarlane, Ontario, Canada; 1:1000) and Alexa 488 conjugated secondary anti-rabbit IgG antibody (Jackson ImmunoResearch; 1:500) and coverslipped with mounting medium containing DAPI (Sigma-Aldrich). Images were taken at ×20 using a Zeiss Axio Imager M2 with ApoTome.

### Data acquisition and analysis

Recordings were obtained and analyzed using *Spike2* software (Cambridge Electronic Design, Cambridge, UK). Neurograms were generated by averaging 300 sweeps and peaks were considered the maximum value. Data are reported as individual values and means ± s.e.m.

## Results

### Optogenetic vagus nerve stimulation in rats

**Fig. 1:**
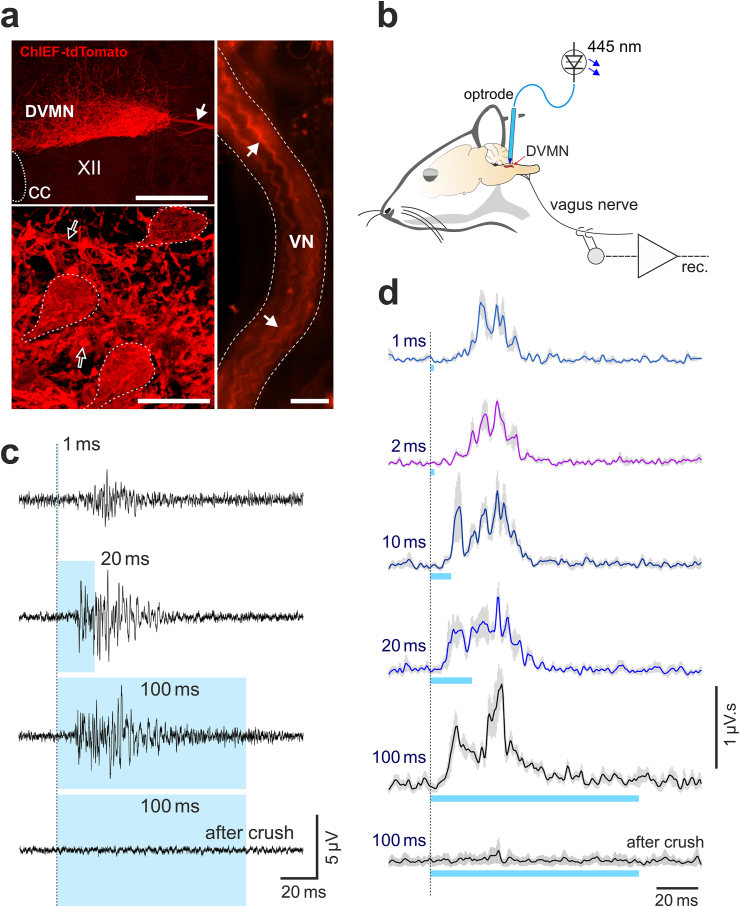
Optogenetic stimulation and properties of vagal preganglionic neurons of the dorsal motor nucleus of the vagus nerve (DVMN) in rats. (**a**) Photomicrographs of the coronal sections of the rat brainstem taken at low (top left) and high (bottom left) magnification illustrating representative examples of ChIEF-tdTomato expression in the DVMN (Bregma level: −13.8 mm). Neurons display specific membrane localization of the transgene expression. The image on the right illustrates DVMN neuronal projections expressing ChIEF-tdTomato visualized in a whole mount preparation of the right cervical vagus nerve. Solid arrows point at the projecting axons of the transduced DVMN neurons; open arrows point at severed neuronal processes within the nucleus; broken while lines outline transduced DVMN neurons. CC, central canal. VN, vagus nerve. XII, hypoglossal motor nucleus. Scale bars: 200 μm (top left), 20 μm (bottom left), 500 μm (right). (**b**) Schematic drawing of the experimental setup in an anaesthetized rat instrumented for stimulation of the DVMN neurons expressing ChIEF-tdTomato by application of blue laser light (via an implanted optrode) and recording of the efferent activity of the vagus nerve at the cervical level. (**c**) Stimulus-triggered averages and (**d**) rectified and smoothed averages (300 sweeps) of the evoked efferent vagus nerve mass action potentials induced by pulses of light of increasing duration delivered at 1 Hz to stimulate the DVMN vagal preganglionic neurons expressing ChIEF-tdTomato (n = 4).

First, in experiments in rats we confirmed that vagal efferent activity can be reliably stimulated by light activation of DVMN neurons expressing ChIEF and characterized the properties of DVMN neurons in this species. DVMN neurons were transduced to express ChIEF-tdTomato (Fig. 1a). Four weeks after the microinjections of the viral vector, the rats were anaesthetized and laser light (445 nm, 1–100 ms pulses) was applied to the dorsal brainstem (Fig. 1b). The electrical activity in the cervical vagus nerve was recorded (Fig. 1c and d). Robust bursts of efferent vagal activity were recorded in response to 1 ms light pulses applied to the DVMN (Fig. 1c and d). Increasing the duration of the laser light pulses led to the increases in the magnitude and duration of the recorded mass action potentials (Fig. 1c and d). The peak of evoked efferent activity was recorded 27 ± 2 ms and 29 ± 2 ms after the onset of the 1 and 2 ms light pulses, respectively, indicating that short pulses activate DVMN neurons with axons conducting at ∼0.8 m s^−1^. Longer light pulses (10, 20, 100 ms) evoked two peaks of efferent activity occurring 12 ± 1 ms and 31 ± 1 ms after the pulse onset (Fig. 1c and d), suggesting that in rats, the longer light stimuli also activate DVMN neurons with axons conducting at ∼2 m s^−1^.

### Expression of ChIEF-tdTomato by DVMN neurons in sheep

**Fig. 2:**
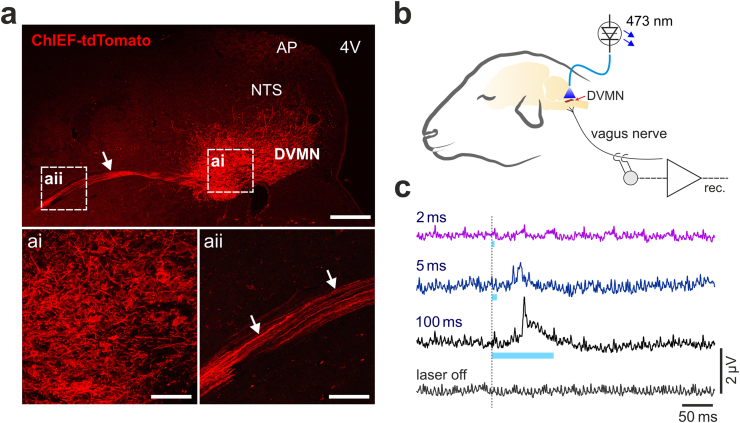
Viral transduction and optogenetic stimulation of the DVMN vagal preganglionic neurons in sheep. (**a**) Photomicrographs of coronal sections of the sheep brainstem taken at low (top) and high (bottom) magnification illustrating representative examples of ChIEF-tdTomato expression in the DVMN. Arrows point at the projecting axons of the transduced DVMN neurons. 4V, fourth ventricle. AP, area postrema. NTS, nucleus of the solitary tract. Scale bars: 400 μm (top), 100 μm (ai, aii). (**b**) Schematic drawing of the experimental setup in an anaesthetized sheep instrumented for stimulation of the DVMN neurons expressing ChIEF-tdTomato by application of blue laser light and recording of the efferent activity of the vagus nerve at the cervical level. (**c**) Stimulus-triggered averages (300 sweeps) of the evoked efferent vagus nerve mass action potentials induced by pulses of light of increasing duration delivered at 1 Hz to stimulate the DVMN neurons expressing ChIEF-tdTomato in sheep.

Experiments in sheep (n = 6) were conducted 12 ± 2 weeks after the injections of the same viral vector into the left DVMN. Strong expression of ChIEF-tdTomato was observed in the DVMN neurons along the mid to rostral axis of the nucleus (Fig. 2a), as well as in the axons of these neurons projecting ventrolaterally through the brainstem (Fig. 2aii). The expression of ChIEF-tdTomato was mostly confined to the population of the DVMN vagal preganglionic neurons as no tdTomato fluorescence was observed in the neighbouring nucleus of the solitary tract or the hypoglossal nucleus (Fig. 2a). Limited transduction was detected in the area postrema, possibly due to some spillover of the vector at the time of the injection. No expression of tdTomato was observed in the neurons of the nodose ganglia, indicating that microinjections of the viral vector into the dorsal brainstem did not result in the transduction of vagal afferents (Supplementary Fig. 1). With viral injections placed into the left DVMN, no transgene expression was observed in the right vagus nerve (Supplementary Fig. 2).

Application of laser light (473 nm) to the dorsal brainstem of an anaesthetized sheep expressing ChIEF-tdTomato in the DVMN neurons triggered mass action potentials, as recorded from the cervical vagus, indicating efferent axonal activation (Fig. 2b and c). Bursts of efferent vagus nerve action potential firing were recorded in response to 5 or 100 ms light pulses applied to the DVMN (Fig. 2c). Light pulses of 2 ms in duration were insufficient to reliably evoke efferent activity.

**Fig. 3:**
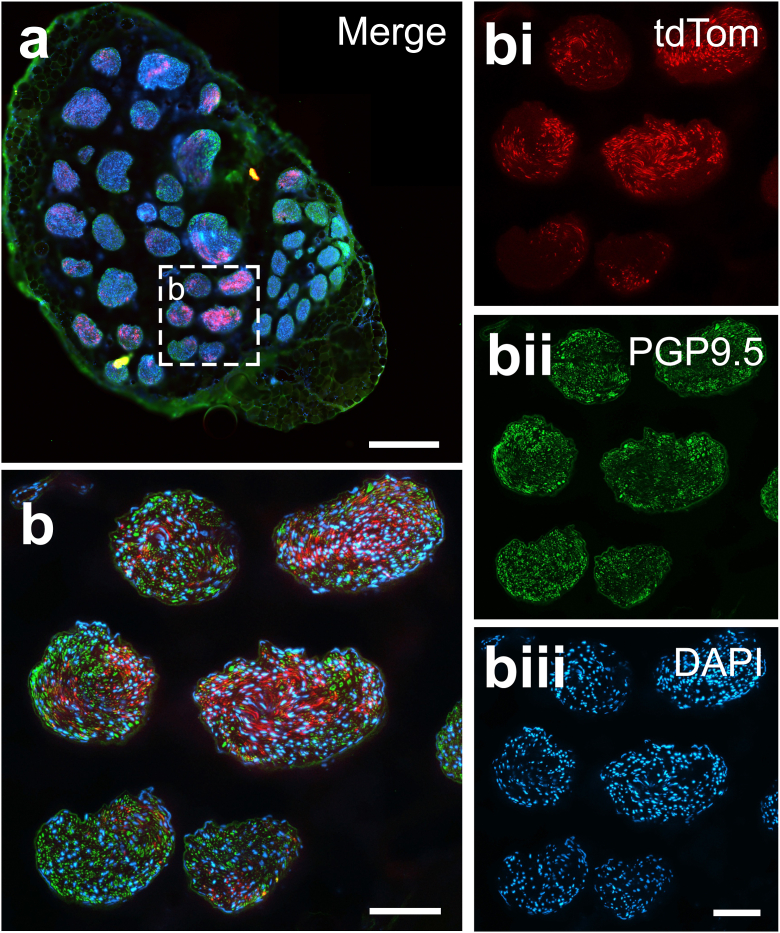
Expression of ChIEF-tdTomato by vagal efferent fibers originating from the DVMN, visualized at the cervical level in sheep. (**a**) Representative cross-section of the whole cervical vagus nerve showing expression of ChIEF-tdTomato (red), Protein Gene Product 9.5 (PGP9.5) immunoreactivity (green) and 4′,6-diamidino-2-phenylindole (DAPI) staining (blue). Scale bar: 500 μm. (**b**) Photomicrograph of six fascicles taken at a higher magnification showing the expression of ChIEF-tdTomato (red), PGP9.5 immunoreactivity (green) and DAPI staining (blue) merged; bi, bii and biii show individual stains. Scale bars: 100 μm. (For interpretation of the references to colour in this figure legend, the reader is referred to the Web version of this article.)

The extent of ChIEF-tdTomato expression in vagal fibers was next examined at different levels of the nerve including the left cervical region, the cardiac branch of the left vagus nerve as well as the thoracic vagus proximal and distal to the cardiac branch (n = 6; representative examples are shown in Fig. 3 and Supplementary Figs. 3–5). Strong expression of tdTomato was observed in efferent fibers at all levels of the vagus nerve examined (Fig. 3; Supplementary Figs. 3–5). The numbers of efferent axons expressing ChIEF-tdTomato varied significantly between different fascicles of the nerve, with some fascicles showing abundant tdTomato fluorescence and some fascicles displaying low, or no, tdTomato fluorescence (Fig. 3).

### Optogenetic vagus nerve stimulation in sheep

**Fig. 4:**
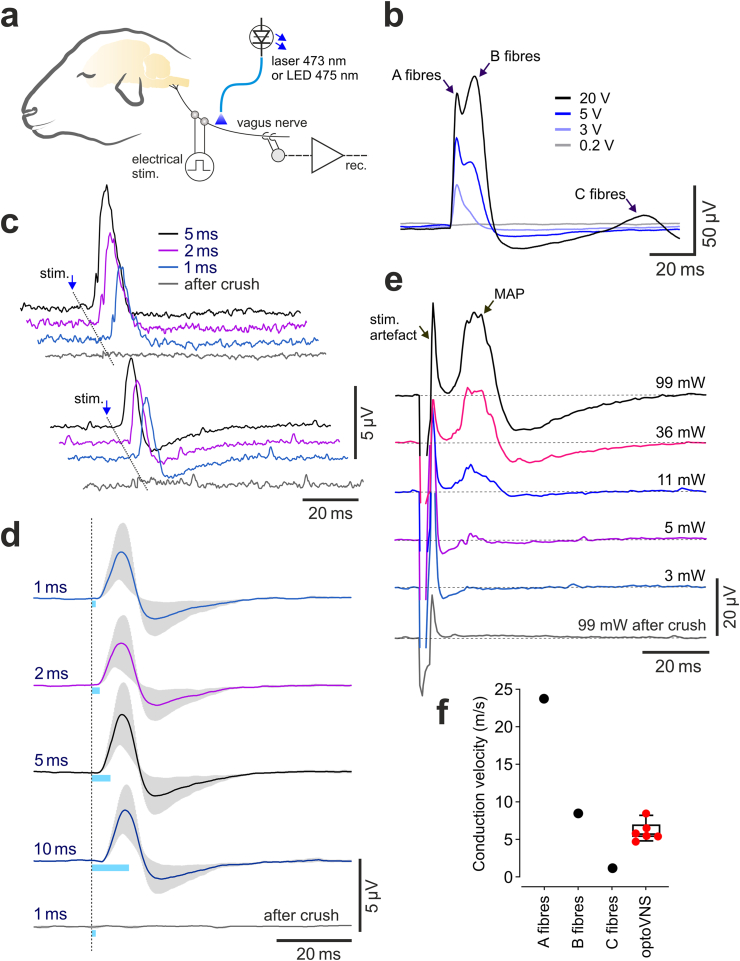
Optogenetic stimulation and properties of vagal efferent fibers originating from the DVMN in sheep. (**a**) Schematic drawing of the experimental setup in an anaesthetized sheep instrumented for electrical or optical stimulation of the nerve and recording of vagal activity at the cervical level. (**b**) Averages of the evoked vagus nerve mass action potentials induced by whole-nerve electrical stimulation illustrating stimulus intensity-dependent recruitment of A, B and C fibres in sheep. (**c**) Representative examples of the recordings obtained in two individual animals and (**d**) group data (n = 4), illustrating stimulus-triggered averages of the evoked efferent vagus nerve mass action potentials, induced by pulses of laser light of increasing duration delivered to the nerve at the cervical level, proximal to the recording site. (**e**) Stimulus-triggered averages of the evoked efferent vagus nerve mass action potentials (MAP) induced by pulses of LED light delivered from a variable distance to the nerve in order to determine the light power threshold, required for activation of the transduced fibers. Light powers indicated are in mW per mm^2^. (**f**) Indicative values (black symbols) denoting the mean conduction velocities that define A, B and C vagal fibres and the group data illustrating the calculated velocities of action potential propagation, recorded in vagal efferent fibers originating from the DVMN in sheep (red symbols, n = 6).

To benchmark light-activation of vagal efferents in sheep, we first characterized electrical properties of vagal fibers at the cervical level in this species. As expected, it was found that vagal fibers propagate action potentials with velocities between ∼1 and 25 m s^−1^ (Fig. 4b,f). Vagal A-fibres had a threshold of activation of 0.3 V and mean conduction velocity of 23.8 m s^−1^, B-fibres had a threshold of activation of 4 V and mean conduction velocity of 8.5 m s^−1^, and for C-fibres these values were 6 V and 1.2 m s^−1^, respectively (Fig. 4b,f).

Blue laser (473 nm) or LED (475 nm) light, applied to the cervical vagus nerve of anaesthetized sheep (n = 6) which were transduced to express ChIEF by the DVMN neurons, triggered robust bursts of efferent activity, as recorded 55–70 mm distal to the site of light application (Fig. 4c–e). The mean calculated conduction velocity of vagal efferent fibers was 6.1 ± 0.5 m s^−1^ (n = 6; Fig. 4f). Varying the light pulse duration between 1 and 10 ms had little effect on the magnitude of the evoked efferent vagus nerve mass action potentials (Fig. 4c and d). Pulses of light delivered by the LED applied to the nerve at the cervical level evoked bursts of efferent activity in the vagal branch within the thoracic cavity (Supplementary Fig. 6).

Light stimulation was next delivered to the whole vagus nerve with the LED positioned at distances of 0 (LED touching the nerve), 1, 3, 5 and 7 mm from the nerve, such that the intensity of light applied to the nerve was systemically varied in order to determine the light power intensity threshold required for stimulation of the transduced fibers (Fig. 4e). The light intensity measurement and calculation are provided in the Supplementary Text and Supplementary Table 1. Robust bursts of efferent activity were induced by pulses of LED light applied with 11 mW mm^−2^ intensity (Fig. 4e). Stimulations with light intensities of 5 mW mm^−2^ or less were insufficient to trigger action potential firing in vagal axons expressing ChIEF (Fig. 4e).

## Discussion

This study demonstrates the feasibility of viral vector targeting of vagal motoneurons to express light-sensitive ion channels and local delivery of light for specific stimulation of vagal efferent activity in large animals. The data show that light stimulation can be applied to the projecting axons of the transduced neurons at a significant distance from the site of the viral vector delivery. To the best of our knowledge, this is the first successful application of an optogenetic technique to selectively stimulate specific neuronal projections within a large mixed nerve, where the transduced axons constitute only a small proportion of all fibers. We show that optoVNS can be applied to stimulate vagal efferents at the cervical level, - the usual site at which the VNS is applied in humans. The ability to stimulate a select group of efferent fibers circumvents the limitations imposed when using electrical VNS where high stimulation intensities are required to activate efferent nerve fibers, invariably also recruiting afferent fibers, and where discrimination between the groups of fibers in a mixed nerve is impossible to achieve. Therefore, this study represents an important step forward in the development of optogenetic technology for targeted autonomic neuromodulation in future experimental pre-clinical studies and eventually in clinical use. Our recent study demonstrated that an analogous approach involving direct optogenetic stimulation of DVMN neurons effectively preserves left ventricular function in experimental model of heart failure in rats [38].

We show that sheep vagal motoneurons can be effectively transduced to express a gene of interest (in this study the optogenetic actuator ChIEF) under the control of the PRSx8 promoter. Similar to the profile of PRSx8-driven expression of transgenes in the brainstem achieved in rodents [[26], [27], [28], [29]], we found that the DVMN neurons in sheep are highly sensitive to the PRSx8-bearing vectors. In sheep, ChIEF-tdTomato expression was strongest in the DVMN with only sparse labelling observed in the adjacent areas of the dorsal brainstem.

Crucial to our ability to stimulate targeted neurons peripherally, the light-sensitive ChIEF channel fused with the fluorescent protein tdTomato was eventually inserted into the membranes along the whole length of the DVMN neuronal axons. We did not perform dedicated experiments to determine the minimum time period required for ChIEF-tdTomato expression to reach the cervical level. Twelve weeks after the microinjections of the viral vector into the DVMN, strong tdTomato fluorescence was observed in the vagal fibers at the cervical level, in the cardiac branch of the vagus nerve and in the thoracic vagus distal to the heart, reaching the level of the diaphragm (∼0.3–0.5 m from the brainstem). One of the animals was used in the experiment nine weeks after the vector microinjections, therefore, nine weeks time period appears to be sufficient to establish robust expression of light-sensitive channels in vagal efferent projections at the cervical level. This indicates that the axonal membrane expression of ChIEF-tdTomato propagates faster than 4.5 mm per day. Membrane proteins such as ChIEF are delivered to the plasma membrane inserted into the walls of vesicles after processing in the Golgi apparatus. The mechanisms of targeted traffic of ChIEF or analogous optogenetic proteins along the axons remain unclear and it is assumed that they reach the distal axonal regions by lateral diffusion. However, this issue requires future clarification.

Pulses of light applied to the whole cervical vagus nerve in sheep triggered bursts of vagal efferent action potentials that propagated at speeds of ∼6 m s^−1^. Robust activations of vagal efferent fibers were observed with 1–2 ms long pulses of light and required LED light power of >5 mW mm^−2^. These data indicate that in sheep, the projections of the transduced DVMN vagal preganglionic neurons have myelinated B fibre axons. Thus, it appears (perhaps not surprisingly) that the DVMN neuronal axons of large animals can conduct much faster than those of smaller mammals (e.g. rats), in which the majority of vagal preganglionic neurons located in the DVMN have C fibre axons, as reported previously [39] and confirmed by the results of this study.

The method of targeted optogenetic stimulation of vagal fibers described here may be useful for functional anatomical mapping of the fascicular organization of the cervical vagus and other mixed nerves. Surprisingly, the functional fascicular organization of the vagus nerve in animals and humans remains completely unknown, despite the widespread experimental and clinical applications of the whole-nerve electrical VNS in conditions including refractory epilepsy, heart failure, depression, and inflammation. A detailed understanding of the fascicular organization of the vagus nerve in large species is important for further development of the VNS technology to allow targeted stimulation of a select group or subset of vagal fibers. Such an understanding may extend our ability to discriminate between fiber subsets beyond afferent and efferent to include function- and organ-specific projections for targeted stimulation at the cervical level.

In summary, this study demonstrates that in species with a large diameter, multi-fascicled vagus nerve, it is possible to selectively stimulate a specific group of nerve fibers using light. Recent advances in human gene therapy map the way toward eventual clinical applications of the optogenetic technology. Next generations of viral vectors may allow targeted transduction of desired populations of vagal fibers projecting to or from individual organs using vector delivery directly into the vagal trunk, for example under ultrasound guidance. OptoVNS can then be applied at the cervical level in order to stimulate or block the activity of specific fibers with unparalleled precision and specificity.

## Funding

This work was supported by the 10.13039/501100000274British Heart Foundation (Refs: RG/14/4/30736 and RG/19/5/34463) and 10.13039/501100000265Medical Research Council (Ref: MR/R01213X/1). A.V.G. is a 10.13039/100010269Wellcome Trust Senior Research Fellow (Ref: 095064). D.M., S.C.M. and Q.B. are supported by the Hugo Charitable Trust. L.C.B, S.T.Y and C.N.M are supported by 10.13039/501100000925NHMRC (Ref: 1128108). S.K. is supported by 5–100 programme. S.T.Y. is an 10.13039/501100000923Australian Research Council Future Fellow (Ref: FT170100363).

L.C.B., S.T.Y., A.K., D.G.S.F., S.G.H., S.J.M., W.S.K., S.M., P.L., G.L.A., R.M.M., C.N.M. and A.V.G. performed research; D.M., Q.B., S-J.G. and S.C.M. designed the LED for light stimulation; A.A.C, A.G.T., S.K. and A.M.A designed the viral vectors; L.C.B., S.T.Y., A.K., W.S.K. and A.V.G analyzed the experimental data; L.C.B., S.T.Y., C.N.M. and A.V.G. wrote the paper. All authors revised the article critically for important intellectual content.

## Declaration of competing interest

All authors declare no conflict of interest.
