# Supplementary material for: Selective optogenetic stimulation of efferent fibers in the vagus nerve of a large mammal
Source: Brain Stimul. 2021 Jan-Feb;14(1):88–96. doi: 10.1016/j.brs.2020.11.010 (PMC7836098; doi:10.1016/j.brs.2020.11.010)
Supplement: Multimedia component 1 [file mmc1.docx]

**Supplementary Information**

*Selective optogenetic stimulation of efferent fibers in the vagus nerve of a large mammal*

Booth, Yao et al.

**Table of Contents:**

**Supplementary Figure 1|** Left nodose ganglion in sheep.

**Supplementary Figure 2|** Right vagus nerve in sheep.

**Supplementary Figure 3|** Expression of ChIEF-tdTomato by vagal efferent fibers originating from the DVMN, visualized at the thoracic level proximal to the cardiac branch in sheep.

**Supplementary Figure 4|** Expression of ChIEF-tdTomato by vagal efferent fibers originating from the DVMN, visualized in the cardiac branch of the left vagus nerve in sheep.

**Supplementary Figure 5|** Expression of ChIEF-tdTomato by vagal efferent fibers originating from the DVMN, visualized in the left thoracic vagus nerve, distal to the cardiac branch in sheep.

**Supplementary Figure 6|** Light stimulation applied at the cervical level triggers mass action potentials in the thoracic vagus nerve in sheep.

**Supplementary Text.** LED light intensity measurements and calculations.

**Supplementary Table 1|** Distance from the nerve vs LED light intensity.

**Supplementary Figure 1|** Left nodose ganglion in sheep. Representative cross-section of the whole left nodose ganglion showing no expression of ChIEF-tdTomato. Scale bar: 500 µm.


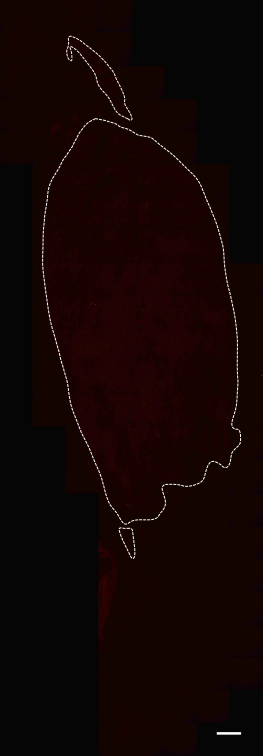


**Supplementary Figure 2|** Right vagus nerve in sheep. A, Representative cross-section of three fascicles showing no expression of ChIEF-tdTomato (tdTom; red) in the right vagus nerve. B, Protein Gene Product 9.5 (PGP) immunoreactivity (green) and C, 4′,6-diamidino-2-phenylindole (DAPI) staining (blue). D, merged photomicrograph. Scale bar: 100 µm.

**Supplementary Figure 3|** Expression of ChIEF-tdTomato by vagal efferent fibers originating from the DVMN, visualized at the thoracic level proximal to the cardiac branch in sheep. A, Representative cross-section of the whole left thoracic vagus nerve showing expression of ChIEF-tdTomato (red), Protein Gene Product 9.5 immunoreactivity (green) and DAPI staining (blue). Scale bar: 500 µm. Ai, B and C, Photomicrographs of five fascicles taken at a higher magnification showing the expression of ChIEF-tdTomato (B; red), PGP9.5 (C; green) and merged (Ai). Scale bars: 100 µm.

**Supplementary Figure 4|** Expression of ChIEF-tdTomato by vagal efferent fibers originating from the DVMN, visualized in the cardiac branch of the left vagus nerve in sheep. A, Representative cross-section of the cardiac branch of the vagus nerve showing expression of ChIEF-tdTomato (red), Protein Gene Product 9.5 immunoreactivity (green) and DAPI staining (blue). Scale bar: 500 µm. B-D, Photomicrographs of fascicles taken at a higher magnification showing the expression of ChIEF-tdTomato (red) and PGP9.5 immunoreactivity (green). Scale bars: 100 µm.

**Supplementary Figure 5|** Expression of ChIEF-tdTomato by vagal efferent fibers originating from the DVMN, visualized in the left thoracic vagus nerve, distal to the cardiac branch in sheep. A, Representative cross-section of the left thoracic vagus nerve, distal to the cardiac branch, showing expression of ChIEF-tdTomato (red), Protein Gene Product 9.5 immunoreactivity (green) and DAPI staining (blue). Scale bar: 500 µm. B-D, Photomicrographs of fascicles taken at a higher magnification showing the expression of ChIEF-tdTomato (red) and PGP9.5 immunoreactivity (green). Scale bar: 100 µm.

**Supplementary Figure 6|** Light stimulation applied at the cervical level triggers mass action potentials in the thoracic vagus nerve in sheep. Stimulus-triggered averages (300 sweeps) of the evoked efferent vagus nerve mass action potentials (MAP) induced by pulses of light delivered by LED (5 ms) to the cervical vagus and recorded from the cervical vagus nerve (~70 mm from the stimulation site) or from the vagus nerve within the thoracic cavity (~200 mm from the stimulation site).


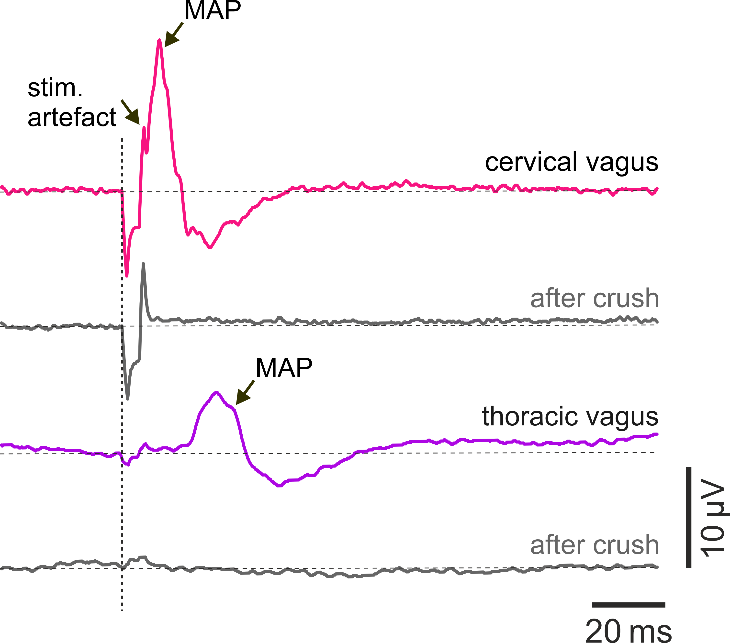


**Supplementary Text.** LED light intensity measurements and calculations.

Light intensity from the LED (Cree XPE2 Blue 475 nm) was measured with an optical power sensor (Thorlabs S170C), connected to a power meter (Thorlabs PM100D). The sensor was mounted directly above the LED (*viz.* on axis) at a distance of 108.5 mm, parallel to the emitting face. This distance is large enough compared to the dimensions of the emitting area of the LED (approximately 2 mm x 2 mm) to treat the LED as a point source for the following calculations.

The amount of light falling on the sensor depends on the solid angle subtended by the sensor, with the LED at the apex. This depends on the distance of the sensor from the LED and the detecting area of the sensor.

The equation to find a solid angle in spherical coordinates is given by:

$$dΩ=\sin\left( \theta\right)d\theta d\varphi$$

Where theta is the polar angle, with ϴ = 0 at the azimuth. The sensor used was square, with a detecting area of 324 mm^2^ (18 mm x 18 mm). By considering it as a circle with equivalent area, the equation can be used as written above, without needing to transform the coordinate system. The radius of a circle with equivalent area is given by:

$$r= \sqrt{\frac{324}{\pi}}=10.16 mm$$

The value of theta to use in the solid angle equation can be found by:

$$\theta=\sin^{-1}\left( \frac{r}{108.5} \right)=5.37^{\circ}$$

The solid angle subtended by the sensor is then given by:

$$Ω=2\pi\int_{0^{\circ}}^{5.37^{\circ}} \sin\left( \theta\right)d\theta$$

$$Ω=2\pi[-cos(\theta)]_{0^{\circ}}^{5.37^{\circ}}$$

$$Ω=2\pi\left[ (-\cos\left( 5.37^{\circ} \right))-(-\cos\left( 0^{\circ}) \right) \right]$$

$$Ω=2\pi\left[ 1-\cos\left( 5.37^{\circ} \right) \right]$$

$$Ω=0.027583 Sr$$

A Tektronix signal generator (AFG1022) and Cleverscope power amplifier (CS1070) were used to power the LED. It was driven at 10 Hz with a 10 % duty cycle. With these settings, the power per pulse can be found by multiplying the power meter reading by ten. The intensity in mW/Sr is then found by dividing the power per pulse by the solid angle subtended by the sensor.

| Pulse current (mA) | Power meter reading (mW) | Power per pulse (mW) | Intensity (mW/Sr) |
| --- | --- | --- | --- |
| 9.6 | 0.015 | 0.15 | 5.45 |
| 112 | 0.17 | 1.7 | 61.77 |
| 148 | 0.22 | 2.2 | 79.94 |
| 196 | 0.28 | 2.8 | 101.74 |
| 240 | 0.34 | 3.4 | 123.54 |
| 288 | 0.39 | 3.9 | 141.70 |
| 344 | 0.45 | 4.5 | 163.50 |
| 396 | 0.51 | 5.1 | 185.30 |
| 456 | 0.57 | 5.7 | 207.10 |
| 512 | 0.63 | 6.3 | 228.90 |
| 576 | 0.68 | 6.8 | 247.07 |
| 636 | 0.74 | 7.4 | 268.87 |
| 696 | 0.79 | 7.9 | 287.04 |
| 768 | 0.84 | 8.4 | 305.21 |
| 840 | 0.89 | 8.9 | 323.37 |
| 904 | 0.94 | 9.4 | 341.54 |
| 968 | 0.99 | 9.9 | 359.71 |
| 1000 | 1.01 | 10.1 | 366.97 |

Excel was used to fit a quadratic equation to the intensity as a function of current (R² = 0.99951):

$$intensity\left( \frac{mW}{Sr} \right)=0.000153884247238306\times I^{2} + 0.511039001785096\times I + 5.76724227607846$$

A geometric model was then used to calculate the size and intensity of spots of light emitted by the LED as the distance from the LED to the tissue increases. Based on the emission profile from the datasheet, the intensity of light emitted by the LED was assumed to be uniform within 30 degrees of the azimuth. The radius of the LED lens was 1.53 mm, this is included in calculations of the spot size:

The diameter of the spot is given by:

$$D_{spot}=2\times\left( d_{tissue}+1.53 \right)\times tan(30^{\circ})$$

The area of the spot is given by:

$$A_{spot}=\frac{{\pi D_{spot}}^{2}}{4}$$

The irradiance of light is assumed to be uniform across the spot (see above), and is given by:

$$irradiance \left( \frac{mW}{{mm}^{2}} \right)= intensity\left( \frac{mW}{Sr} \right)\times solid angle (Sr)\div area({mm}^{2})$$

The solid angle subtended within 30° of the azimuth is given by:

$$Ω=2\pi\left[ 1-\cos\left( 30 \right) \right]$$

$$Ω=0.841787 Sr$$

Using this series of equations, the diameter of a uniform intensity spot of light can be calculated for arbitrary distances between the tip of the LED and the tissue, for driving currents up to 1000 mA. In this study, the LED was driven at 700 mA, with distances from the nerve of 0, 1, 3, 5, and 7 mm.

**Supplementary Table 1|** Distance from the nerve *vs* LED light intensity. Values were calculated with a geometric model of light transmission, with the assumption that the light is emitted by the LED with uniform intensity within a viewing angle of 30°, and that the illuminated spot is on a flat surface. The reported spot area and irradiance are for light within this viewing angle, outside of this angle the irradiance is non-uniform and decreases rapidly with increasing angle.

| Distance from the LED tip (mm) | Spot diameter (mm) | Spot area (mm^2^) | Spot irradiance  (mW mm^-2^) |
| --- | --- | --- | --- |
| 0 | 1.77 | 2.45 | 98.93 |
| 1 | 2.92 | 6.70 | 36.18 |
| 3 | 5.23 | 21.49 | 11.29 |
| 5 | 7.54 | 44.65 | 5.43 |
| 7 | 9.85 | 76.20 | 3.18 |
